# Metascience in Bioinformation

**DOI:** 10.6026/97320630016004

**Published:** 2020-01-01

**Authors:** Allen Khakshooy, Quyen Bach, Vandan Kasar, Francesco Chiappelli

**Affiliations:** 1 Technion, Faculty of Medicine, Israel Institute of Technology, Isreal; 2 Dental School, AT Still University, USA; 3 Dental School, University of California San Francesco, USA; 4 Center for the Health Sciences, University of California Los Angeles, USA

## Abstract

Metascience refers to the systematic process that uncovers, builds, evaluates, organizes and disseminates scientific advances. It is the principal tool at the disposal of the society to combat the debilitating effects of “false information” on health related data and its constituents.

## Background

The roots of Western science are to be found in the philosophers of antiquity, and their observations of the matter, energy, phenomena, and the interaction between them, which together constituted their surrounding world. They called it the study of nature, or physics. Soon, they expanded their inquisitive horizon to the identification of what lied behind, or above the physical forces of nature; that is, what was "meta"-physical. With the centuries, metaphysics broke new frontiers in characterizing the fundamental nature of reality, the relationship between mind and matter, between substance and attribute, and between potentiality and actuality. But the prefix "meta" continued to be used to indicate the study of the principles that drive a given domain of inquiry. Case in point, modern cognitive psychology has two principal domains: the study of cognition and memory, and the study of the processes that drives and determines cognition and memory, and that are de facto "above" cognition and memory in se, that is meta-cognition. In brief, meta cognition is cognition about cognition, thinking about thinking, awareness of higher intellectual skills and processes.

In the same vein, science can be defined as the systematic enterprise that uncovers, builds, organizes and disseminates knowledge by means of testable explanations, analyses and predictions about our psycho-physiological nature and the environment that surrounds us. Metascience is, by extension, our scientific inquiry into science itself. Metascience [1] is the systematic enterprise that uncovers, builds, organizes and disseminates formative and summative evaluative knowledge about scientific advances by means of testable explanations, analyses and predictions [2]. Now more than ever, it is imperative that we as a conscientious species establish a universal system that empowers us all with scientific information that is accurate and reliable.

## Methods, Analyses, Evaluation and Replicability:

In the last two decades, since the establishment of evidence-based medicine and evidence-based dentistry, metascience has developed, tested, verified and established a stringent methodological strategy [3].For every question under study, a research question is crafted following rigid criteria that define inclusion and exclusion boundaries. The resulting statement is validated through an analytical framework uniquely designed for each research question.

This process results in the generation of certain key words and phrases that aid in pinpointing the exact scientific evidence that pertains to the research question. The pertinence of each uncovered report to the research question is then verified by two independent assessors. The resulting short-list of evidentiary documentation –the "bibliome"–, which is directly and uniquely pertinent to the research question, is evaluated for the level and quality of the evidence it purports to present. In brief, the level of the evidence refers to the type of study - cf. observational correlative, such as the recent report that hair dyeing seems to be associated with breast cancer risk; observational longitudinal, in which patients serve as their own control, or clinical trials, the highest producible "level of evidence". By contrast, the quality of the evidence refers to the nature of the data obtained, the structure of the sample that generated the data, the validity and reliability of the measuring instruments that produced the data, and the exactness of the statistical analysis of the data.

One of great utilities of statistical analysis is its ability to dive within the information to elucidate the truth principally by minimizing that which is erroneous [5]. The "best" reports are obtained by means of a stringent acceptable sampling statistical analysis, commonly referred to as meta-analysis. The validity of these analyses is repeatedly evaluated by means of formative and summative evaluation, and updated as required, because it is self-evident that scientific research in every field is an active and ongoing process. New reports and information are constantly being produced so that they may confirm or contradict, and certainly expand, current scientific knowledge. Evidence-based medicine and dentistry, and metascience in general relies on the entire body of the "best" and "available" evidence.

Metascience, like all science, is grounded not only on the stringency of its methodology and data analysis, but also of the replicability of its findings. Systematic reviews of the research literature as outlined above are the core of metascience, and must be both peer-reviewed before dissemination, and replicated before conclusions are taken as "the best available" evidence in support or in refuting the original research hypothesis.

## Reporting and Dissemination:

Metascience is disseminated by several means and at several levels [4]. Formal systematic reviews are peer-reviewed and published in specialized academic journals. These are typically long and complex technical reports, rich in methodology details that permit replication, arduous statically analyses and meta-analyses, and interpretations of clinical effectiveness conclusions. Critical summaries of systematic reviews are shorter documents that typically do not exceed two-to-three printed pages. They summarize the meta scientific research protocol and its findings, and outline the implications for clinical effectiveness. They are written by and for experts in the field in meta scientific jargon, published in peer-reviewed journals, and aid the clinician to optimize evidence-based patient-centered recommendations.

However, neither systematic reviews nor critical summaries serve to disseminate the "best available" evidence to the patient, stakeholders, and general lay population. To achieve that most critical step in metascience dissemination, it is timely and critical "to translate" the metascientific jargon into lay language. This is an important pillar of the greater, translational model of healthcare, which is the inevitable future. The ease of communication and understanding plays a critical role in services that aid a patient-centered approach, such as telehealth and its encompassing applications [4-6].

Today, the weakest step in the metascience endeavoris its translation to stakeholders. Scientists willing and capable of translating systematic reviews or critical summaries into easy-reading documents that coherently reflect the stringency and validity of the reported "best available" evidence are few and far between. But, no system of validation for such translations has yet been developed, standardized, validated and widely accepted in the field.

## Conclusion

Metascience involves the use of new and stringent scientific methodology to study science itself for raising the overall quality of scientific knowledge, and of ensuring the highest possible effectiveness in its patient-centered clinical applications. Metascience is research on research: the science of science in all fields from astrophysics and psychology to molecular biology; from the social and political sciences to the health sciences. In the context of health care, metascience is often referred to as evidence-based medicine/dentistry/nursing, and seeks primarily to ensure the highest possible effectiveness of evidence-based treatment in a personalized patient-centered model of clinical intervention. This translational model of healthcare stresses active involvement of the patient and stakeholders in the clinical-decision making process, and is therefore anchored in the optimization of precise and accurate, factual evidence [6].

The very goal of metascience is to ensure that scientific progress and information grow from accurate, systematically verified, statically incontrovertible, and unquestionably true facts. It is no longer enough for our quests of minimizing false information to be limited to misinformation, i.e., inaccurate information that was made by honest error. Now, we must lend a keen eye to and actively search out disinformation – inaccurate information used intentionally to deceive. Metascience is the principal tool we have now, and must continue to develop and strengthen in the next decade to combat the undermining effects of "false information" in science in general - cf. creationism vs. evolution theory -, or in the health sciences specifically - cf., root canals cause breast cancer; anti-vaccination movement, etc. Surely, there are few greater dangers than scientific information - which is, in effect, information for the ultimate benefit of society - that is falsified, whether intentionally or not.

## About the authors:

Allen Khakshooy is currently a medical pre MD student. Ms. Quyen Bach and Vandan Kasar are currently dental pre D.D.S. students.
